# Mineral Ecology: Surface Specific Colonization and Geochemical Drivers of Biofilm Accumulation, Composition, and Phylogeny

**DOI:** 10.3389/fmicb.2017.00491

**Published:** 2017-03-28

**Authors:** Aaron A. Jones, Philip C. Bennett

**Affiliations:** Department of Geological Sciences, University of Texas at AustinAustin, TX, USA

**Keywords:** biofilms, microbial communities, cave microbiology, subsurface, bioreactors, microbe/mineral interactions

## Abstract

This study tests the hypothesis that surface composition influences microbial community structure and growth of biofilms. We used laboratory biofilm reactors (inoculated with a diverse subsurface community) to explore the phylogenetic and taxonomic variability in microbial communities as a function of surface type (carbonate, silicate, aluminosilicate), media pH, and carbon and phosphate availability. Using high-throughput pyrosequencing, we found that surface type significantly controlled ~70–90% of the variance in phylogenetic diversity regardless of environmental pressures. Consistent patterns also emerged in the taxonomy of specific guilds (sulfur-oxidizers/reducers, Gram-positives, acidophiles) due to variations in media chemistry. Media phosphate availability was a key property associated with variation in phylogeny and taxonomy of whole reactors and was negatively correlated with biofilm accumulation and α-diversity (species richness and evenness). However, mineral-bound phosphate limitations were correlated with less biofilm. Carbon added to the media was correlated with a significant increase in biofilm accumulation and overall α-diversity. Additionally, planktonic communities were phylogenetically distant from those in biofilms. All treatments harbored structurally (taxonomically and phylogenetically) distinct microbial communities. Selective advantages within each treatment encouraged growth and revealed the presence of hundreds of additional operational taxonomix units (OTU), representing distinct consortiums of microorganisms. Ultimately, these results provide evidence that mineral/rock composition significantly influences microbial community structure, diversity, membership, phylogenetic variability, and biofilm growth in subsurface communities.

## Introduction

It is estimated that up to 99.9% of microbial biomass in a subsurface environment is attached to surfaces as biofilms (Madigan et al., 2009). Microbial attachment to natural surfaces is a complex and dynamic process involving interaction between the organism, the surface, and the aqueous phase. The subsurface is a complex and heterogeneous environment where varying mineralogy results in different surface chemistries and microscale spatial heterogeneity that contribute to growth, present challenges, or influence community membership. The purpose of this study is to characterize the mineralogical contribution to microbial diversity, investigating the contribution of different natural surface types under a range of environmental conditions.

Recent studies suggest a link between mineral composition and colonization by specific microbial communities. Factors controlling dynamic diversity, growth, and specific survival strategies include and have greater implications for adjacent studies of physical properties (hydrodynamics) of bulk fluid (Kugaprasatham et al., 1992), physicochemical nature of surfaces (Dalton et al., 1994; Rogers et al., 1998; Rogers and Bennett, 2004; Carson et al., 2009; Sylvan et al., 2012), microbial community composition (Lawrence et al., 1991), and nutrient cycling and availability (primarily carbon, phosphorous, and nitrogen; Ohashi et al., 1995; Huang et al., 1998; Rogers et al., 2001). pH is often identified as a key factor controlling microbial community composition (Fierer and Jackson, 2006; Lauber et al., 2009; Chu et al., 2010; Siciliano et al., 2014; Winsley et al., 2014). Biofilm and extracellular polysaccharides (EPS) production are both influenced by the nutrient content of the growth medium with respect to available carbon, or phosphate limitations (Ellwood et al., 1982; Matin et al., 1989; Wrangstadh et al., 1990). However, few studies examine complex natural communities on natural surfaces. In oligotrophic environments, such as those in the subsurface, microorganisms are likely highly reliant on minerals (“mineraltrophic”) to support various biogeochemical processes (Stevens, 1997; Anderson, 2001; Chapelle et al., 2002; Edwards et al., 2005, 2012).

Previously, we found that, in oligotrophic conditions, specific guilds showed an affinity for specific mineral types according to their metabolic requirements and environmental tolerances (Jones and Bennett, 2014). Sequences from similar surface types (e.g., carbonates, silicates, aluminosilicates) were more taxonomically similar. Specifically, given a choice of surface types, neutrophilic, but acid-producing sulfur-oxidizers (SOB) were dominant on highly-buffering carbonates, acidophiles on non-buffering silicates, Gram-positives on silicates, and aluminum tolerant bacteria on aluminosilicates (Jones and Bennett, 2014). Additionally, mineral-phosphate availability was correlated with biofilm accumulation (Jones and Bennett, 2014). Those experiments were designed to favor growth of autotrophic SOB and to mimic the nutrient-limited environment found within Lower Kane Cave (WY, USA) where sulfidic water serves as the metabolic backbone for a diverse microbial community (Egemeier, 1981; Engel et al., 2004). However, natural environments are subjected to variable geochemical conditions that may influence microbial surface colonization. We hypothesize that surface type is an important variable influencing biofilm community structure (growth, taxonomic, and phylogenetic variability) even under distinct geochemical conditions.

For this study we utilize high-throughput 454-pyrosequencing (Margulies et al., 2005) of bacterial 16S rRNA sequences to examine the response of community structure and diversity to environmental stimuli (pH variability, carbon and phosphate limitations/amendments) as biofilms develop on different mineral surfaces within flow-through biofilm reactors. This allowed us to assess the major biogeochemical reactions in a controlled setting and further constrain the key parameters affecting microbial diversity, not by replicating the complexity of the natural system, but by fine tuning parameters in order to evaluate their influence on microbial communities. We use phylogenetic distance measures (UniFrac), to account for the relationships among populations attached to the various surfaces (Lozupone and Knight, 2005). We use permutational multivariate analysis of variance (PERMANOVA) to evaluate mineralogical and environmental influence on microbial community structure (McArdle and Anderson, 2001). Additionally, we test our hypothesis on the 16S rRNA sequences and growth data from Jones and Bennett (2014) using a more robust methodology. Taxonomic variations among specific guilds (SOB, SRB, Gram-positives, Acidophiles), are identified to interpret the ecological role of the detected taxa.

## Materials and methods

### Flow through biofilm reactor

We used a modified CDC biofilm reactor (Biosurface Technologies, Bozeman, MT, USA; see http://biofilms.biz/products/biofilm-reactors): a 1-liter glass vessel with a ported polyethylene top that supports 8 polypropylene rods, each holding up to three coupons (12.7 mm OD disks ~3 mm thick). The reactor was operated as a continuous-flow stirred reactor at 1.5 ml/min liquid medium flow. Consistent shear and mixing at all positions within the reactors was maintained using a stir vane rotated by a magnetic stir plate.

The mixed environmental inoculum was collected in the field at Lower Kane Cave (LKC) (WY, USA) in sterile falcon tubes and was identical to that used in Jones and Bennett (2014). The inoculum is a consortium composed primarily of autotrophic sulfur-oxidizing members of lineages *Gammaproteobacteria* (34.7%) of the genus *Thiothrix*, and *Epsilonproteobacteria* (62.4%) of the genus *Sulfurovum* (Engel et al., 2003, 2004; Jones and Bennett, 2014), but with many other Bacterial lineages at lower abundance. Approximately 15 ml of the raw mat (inoculum) was added to the sterilized CDC biofilm reactor for each experiment.

Synthetic cave water was prepared by equilibrating DI-H_2_O water with finely powdered Iceland spar calcite to equilibrium. The solution was filtered to 0.2 μm and 0.1 g MgSO_4_ and 0.25 g NH_4_Cl were added per liter and autoclaved at 121°C for 45 min before adding 2 and 5 ml/L of filter-sterilized trace metal solution and Wolfe's Vitamin solution, respectively (Burlage, 1998). The reduced sulfur electron donor was S_2_${\text{O}}_{3}^{2-}$ prepared from a stock filter-sterilized 1 M solution of Na_2_S_2_O_3_ mixed in-line via a syringe pump to a final concentration of 0.83 mM.

Amendments (P, C, or both) were then added to this basic liquid media or pH adjusted to examine the influence of environmental conditions (Table 1). Specifically, the carbon/phosphorus-limited (CP-Limited) media used in Jones and Bennett (2014) was this basic liquid media with sterile 0.1 N H_2_SO_4_ added to achieve a final pH of 6.9 (Jones and Bennett, 2014). The C-Amended medium was prepared by amending the basic medium with 5 mM Na-Acetate, 5 mM Na-Lactate and filter sterilized 0.1 N H_2_SO_4_ added to a final pH of 6.9. The P-Amended media was prepared with 0.53 g/L KH_2_PO_4_ and 0.12 g/L K_2_H_2_PO_4_ and filter sterilized NaOH added to a final pH of 8.3. The no limitation medium (CP-Amended) was amended with 5 mM Na-Acetate, 5 mM Na-Lactate, 5 mM Na-Formate, 0.53 g/L KH_2_PO_4_, 0.12 g/L K_2_H_2_PO_4_, and 0.1 N H_2_SO_4_ added to achieve a final pH of 6.9 (Table 1).

**Table 1:** **Media recipes for each of the four reactor (treatment) conditions**.

**Composition of Medias for Each Treatment (L**^**−1**^**)**				
**Component**	**CP-Limited**	**C-Amended**	**P-Amended**	**CP-Amended**
Calcite (Eq.) DI	1,000 ml	1,000 ml	1,000 ml	1,000 ml
Na_2_S_2_O_3_	10 mM	10 mM	10 mM	10 mM
MgSO_4_	0.25 g	0.25 g	0.25 g	0.25 g
NH_4_Cl	0.1 g	0.1 g	0.1 g	0.1 g
Trace Metals	2.1 ml	2.1 ml	2.1 ml	2.1 ml
Wolfe's Vitamins	5.3 ml	5.3 ml	5.3 ml	5.3 ml
KH_2_PO_4_	–	–	0.53 g	0.53 g
K_2_H_2_PO_4_	–	–	0.12 g	0.12 g
Na-Lactate	–	5 mM	–	5 mM
Na-Acetate	–	5 mM	–	5 mM
Na-Formate	–	–	–	5 mM
pH initial	6.9	6.9	8.3	6.9
pH reactor	5.7	7.5	7.9	7.8

*−, represents none added*.

### Surface substrata preparation

Mineral/rock substrata were selected to represent the lithology of a variety of geologic environments [carbonates, silicates, aluminosilicates, planktonic (not attached)] (Table 2). Additionally, some advantages and disadvantages to microorganisms equipped to exploit (or defend against) them are described in Table 2. Specimens of calcite, microcline, albite, basalt, and quartz were obtained from Ward's Natural Science Establishment Incorporated. These materials have been previously characterized (Bennett et al., 2001; Jones and Bennett, 2014). Unaltered Mississippian-age Upper Madison Limestone, Upper Madison Dolostone and the contained chert, were collected from an outcrop near Lower Kane Cave. The Madison Limestone is nearly pure calcite (microsparite) with a minor quartz component, and the Madison Dolostone is nearly pure dolomite also with a minor quartz component (Plummer et al., 1990). Mineral/rock coupons were prepared using previously published methods (Jones and Bennett, 2014).

**Table 2:** **Surface types, general compositions, and biogeochemical significance of the rocks/minerals used in these biofilm reactor experimental treatments**.

**Surface type**	**Surface**	**General composition and origin**	**Biogeochemical significance**
Carbonates	Calcite	CaCO_3_ Iceland Spar Calcite	High-Buffering Capacity^b^, No Trace Nutrients
	Madison Limestone	CaCO_3_ Lower Kane Cave, WY, USA	High-Buffering Capacity^b^, Trace Nutrients, High-${\text{PO}}_{4}^{2-}$
	Madison Dolostone	CaMg(CO_3_)_2_ Lower Kane Cave, WY, USA	High-Buffering Capacity, Trace Nutrients, High-${\text{PO}}_{4}^{2-}$
Aluminosilicates	Microcline	KAlSi_3_O_8_ Ontario Microcline^a^	Low-Buffering Capacity, Low-Trace Nutrients^a^, Potentially Toxic Al
	Albite	NaAlSi_3_O_8_ Ontario Plagioclase^a^	Low-Buffering Capacity, Low-Trace Nutrients^a^, Potentially toxic Al
Silicates	Chert	SiO_2_ Lower Kane Cave, WY, USA	Low-Buffering Capacity, Low-Trace Nutrients^a^
	Basalt	Fe, Mg, Ca, Al, Si, O Columbia River Basalt^a^	Low-Buffering Capacity, High-${\text{PO}}_{4}^{2-}$, H_2_ source for Methanogens & ${\text{SO}}_{4}^{2-}$ reducers^c^
	Quartz	99.78% SiO_2_ Hydrothermal Crystal^a^	Low-Buffering Capacity, No-Trace Nutrients^a^

This table is modified from Jones and Bennett (2014). Superscripts refer to papers with additional information

a Bennett et al., 2001;

b *Steinhauer et al., 2010; ^c^Edwards et al., 2005*.

### Biomass measurement and extraction

To measure biomass accumulation after 3 weeks, triplicate mineral coupons were weighed (wet, biofilm attached), dried overnight at 104°C, weighed again (dry, biofilm attached), processed by 3 × 5-min cycles of alternating sonication and vortexing in a calcite equilibrated (to prevent dissolution) 2% tween 20 solution to remove biomass, dried overnight again, and weighed again (dry, biofilm removed). The dry weight of biomass accumulated is the difference between the final dry weight with biomass and the dry weight after processing.

Using these methods, biofilm growth curves were constructed in order to determine the standard duration (3-weeks) of each experiment. Curves were constructed for both pure and mixed culture treatments under CP-Limited, C-Amended, and P-Amended conditions. For these experiments, limestone was the sole surface type occupying all 24 coupon spaces. Two limestone coupons (chosen randomly) were sacrificed at 48-h intervals and biomass was measured according to the method described above. The resulting curves are shown in Supplementary Figure 1.

For DNA extraction, biomass was aseptically isolated from mineral coupons in 1 mM EDTA and 0.9X phosphate-buffered saline (PBS with physical disruption by freeze-thaw (3 times, −80°–65°C) cycles followed by alternating sonication and vortexing (3 × 5-min) (Jones and Bennett, 2014). The biomass was isolated from solution by centrifugation at 5000 rpm for 10 min, and the supernatant decanted. DNA extraction from biomass was conducted using an Ultraclean Microbial DNA Isolation Kit (MoBio Laboratories, Inc.; Catalog # 12224-50). DNA samples were quantified and qualified using a Nanodrop spectrophotometer (Nyxor Biotech, Paris, France). Bacterial tag-encoded FLX-titanium amplicon pyrosequencing (bTEFAP) was used to evaluate the bacterial populations removed from the mineral surfaces at MR DNA Lab (http://www.mrdnalab.com, Shallowater, TX, USA). The bTEFAP procedures are based on Research and Testing Laboratory protocols http://www.researchandtesting.com and are previously described (Dowd et al., 2008). Briefly, the 16S universal Eubacterial primers 27F (5′-AGRGTTTGATCMTGGCTCAG-3′) and 519R (5′-GTNTTACNGCGGCKGCTG-3′) were used to amplify the v1-v3 region of 16S rRNA genes using 30 cycles of PCR. HotStarTaq Plus Master Mix Kit (Qiagen) was used for PCR under the following conditions: 94°C for 3 min, followed by 28 cycles of 94°C for 30 s; 53°C for 40 s and 72°C for 1 min after which a final elongation step at 72°C for 5 min was performed. After PCR, all amplicon products from the different samples were mixed in equal volumes and purified using Agencourt Ampure Beads (Agencourt Bioscience Corporation, Beverly, Ma). Adaptors and barcodes for 454 pyrosequencing were ligated, and sequencing on a Roche 454 GS-FLX Titanium™ (454 Life Sciences, Branford, CT, USA).

Sequences were quality screened prior to clustering into operational taxonomic units (OTUs) using the open source software package QIIME version 1.9 (http://qiime.sourceforge.net; Caporaso et al., 2010). We removed from further analysis: sequences < 200 or >550 bp, sequences with ambiguous base calls (>6 bp), sequences with homopolymer runs (>6 bp), low quality scores (<25), sequences with primer mismatches, and barcode errors (>1 bp). Additionally, noisy sequences were discarded using the “denoise_wrapper” script (Reeder and Knight, 2010). Chimeric sequences were removed using ChimeraSlayer with the QIIME default settings after OTU-picking and taxonomic assignment. The uclust method was used to pick *de novo* OTUs at 3% (genus level) divergences. Representative sequences for each OTU were then aligned with PyNAST and taxonomy was assigned with the uclust consensus taxonomy assigner using the greengenes_13_8 reference database. Potential contaminants and OTUs with < 4 members were then filtered from the resulting OTU table. Assigned taxa were compared with extant taxa described in the literature, at the genus level, to infer the putative ecological role of these microorganisms.

All amplicons obtained from this study were submitted to the NCBI Sequence Read Archive (SRA) under the Bioproject PRJNA323607, with the BioSample accession numbers SAMN05181927- SAMN05181927 (http://www.ncbi.nlm.nih.gov). Amplicon data and SRA accesion numbers for each sample are included in Supplemental Table 2.

### Biodiversity metrics and statistical analysis

Community diversity was evaluated using both standard statistical analysis on OTUs (e.g., Species Richness, Shannon-Wiener, Simpson's) and phylogenetic analysis (Bohannan and Hughes, 2003). α and β-diversity values were calculated on the resulting datasets using QIIME with selective manual calculations for validation (Hill et al., 2003; Caporaso et al., 2010). Due to the different number of sequences among samples, the data was normalized for diversity analysis using rarefaction curves. Rarefaction curves of the observed richness were calculated in QIIME using 100,000-fold resampling without replacement (Supplementary Figure 2). Rarefactions were performed for each treatment independently at the maximum subsampling depth to include all samples within a treatment. Estimates of α-diversity were based on evenly rarefied OTU abundance matrices and included observed species richness (S), the reciprocal of the Simpson dominance index (D), Shannon-Weiner index (H'), and species evenness (E) (Hill et al., 2003). Sampling effort was estimated using Good's coverage (Good, 1953). The reciprocal Simpson dominance index (D) gives more weight to dominant OTUs by expressing the likelihood that two individuals, chosen at random, will belong to different OTUs. The effect of geochemical (pH_in_, pH_out_, C-P Availability) and mineralogical (mineral geochemistry, P-Availability, Buffering Capacity) variations on microbial community diversity was assessed at rarefication depths according to the smallest sample within each subset.

Beta diversity (global diversity) both between communities on multiple surfaces and between treatments was assessed using weighted UniFrac (phylogenetic) distance matrices at sequence divergences of 3% (Lozupone et al., 2011) between samples were calculated after rarefying all samples. The effect of geochemistry on microbial/mineral associations was depicted using UniFrac distance based 3D Principal Coordinate Analysis (PCoA) plots (Borg and Groenen, 2005), and Unweighted Pair Group Method with Arithmetic Mean (UPGMA) trees (Felsenstein, 2004).

To assess the statistical significances in microbial community dissimilarity between particular groups, the UniFrac distance matrix generated in QIIME was analyzed with R statistical software (R foundation for Statistical Computing, Vienna, Austria). Statistical significance was considered at *P* < 0.05. Alpha diversity metrics were compared within and between treatment types using Wilcoxon signed rank test. Testing for the presence of a significant effect of sample type on beta diversity metrics was done using permutational multivariate analysis of variance (PERMANOVA) and the “Adonis” function from the R package “vegan,” which partitions the distance matrix among sources of variation, fits linear models to distance matrices and uses a permutation test (10^5^) with pseudo-F ratios to obtain *P*-values (McArdle and Anderson, 2001). Data sets for the effect of phosphate and carbon on microbial biomass were analyzed by analysis of variance (ANOVA) with significance at *P* < 0.05.

## Results

### Biomass abundance

**Figure 1:**
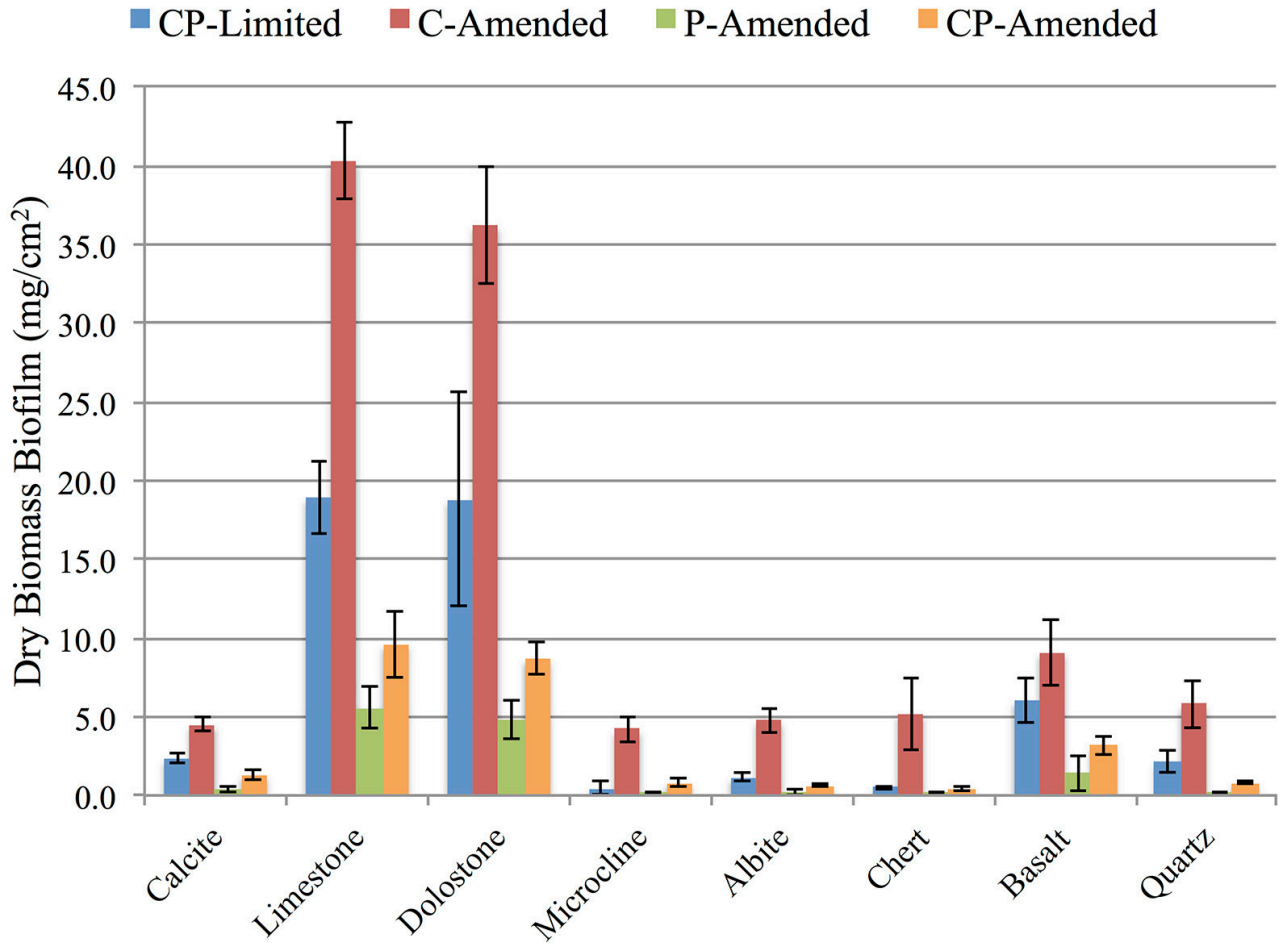
**Dry weight (mg/cm2) of biomass accumulation on surfaces for each reactor treatment**. Error bars denote standard deviation, *n* = 3. See Supplementary Table 1 for values.

Biomass accumulation on each surfaces was measured (in triplicate) after 3-weeks for each treatment. Total dry mass of biofilm on all minerals for the CP-Limited from Jones and Bennett (2014), C-Amended, P-Amended, and CP-Amended treatments was 50.5, 110.1, 12.8, and 25.4 mg·cm^−2^, respectively (Figure 1). ANOVA analysis revealed that the addition of phosphate to the media decreased biofilm biomass (*P* < 0.002), while the addition of available carbon significantly increased biofilm biomass (*P* < 0.04). In all treatments, P-bearing surfaces (limestone, dolostone, and basalt) had significantly higher biomass (Figure 1). In the CP-Limited treatment, high-P surfaces accumulated up to 40X that of low-P surfaces. In the C-Amended treatment was up to 10X higher (Supplementary Table 1). The P- and CP-Amended treatments had the lowest biofilm accumulations on every surface, but even here the high-P minerals accumulated ~3–60X higher biomass than the low-P surfaces. Although this is the largest relative difference, the actual variation in total biomass (SD ~2.3 mg·cm^−2^) was lowest in the P-Amended treatment as most of the biomass was planktonic (Supplementary Table 1).

### Effect of treatment conditions and surface type on bacterial diversity

There were 578,969 total raw sequences obtained from all samples in the four treatments. Of those, a total of 209,973 (CP-Limited—27,173 total with an average of 3397 ± 3133 per sample, C-Amended—66,383 total with an average of 7,376 ± 5,662 per sample, P-Amended—34,847 total with an average of 4205 ± 934 per sample, CP-Amended—78,570 total with and average of 8,730 ± 2,900 per sample) bacterial 16S high-quality sequences with an average read length of 440 bp were obtained for the 35 samples. Rarefaction curves for richness of 33 (except CP-Limited microcline and albite) of the 35 samples approach a plateau at their respective maximum sampling depth, indicating an adequate sampling procedure (Supplementary Figure 2). Additionally, the overall average Good's coverage is 98.4 ± 0.9% including CP-Limited microcline and albite with Good's coverage values of 95.1 and 95.7%, respectively, indicating an adequate sampling procedure (Supplementary Table 2). Changes in diversity due to treatment conditions and surface type were evaluated.

**Figure 2:**
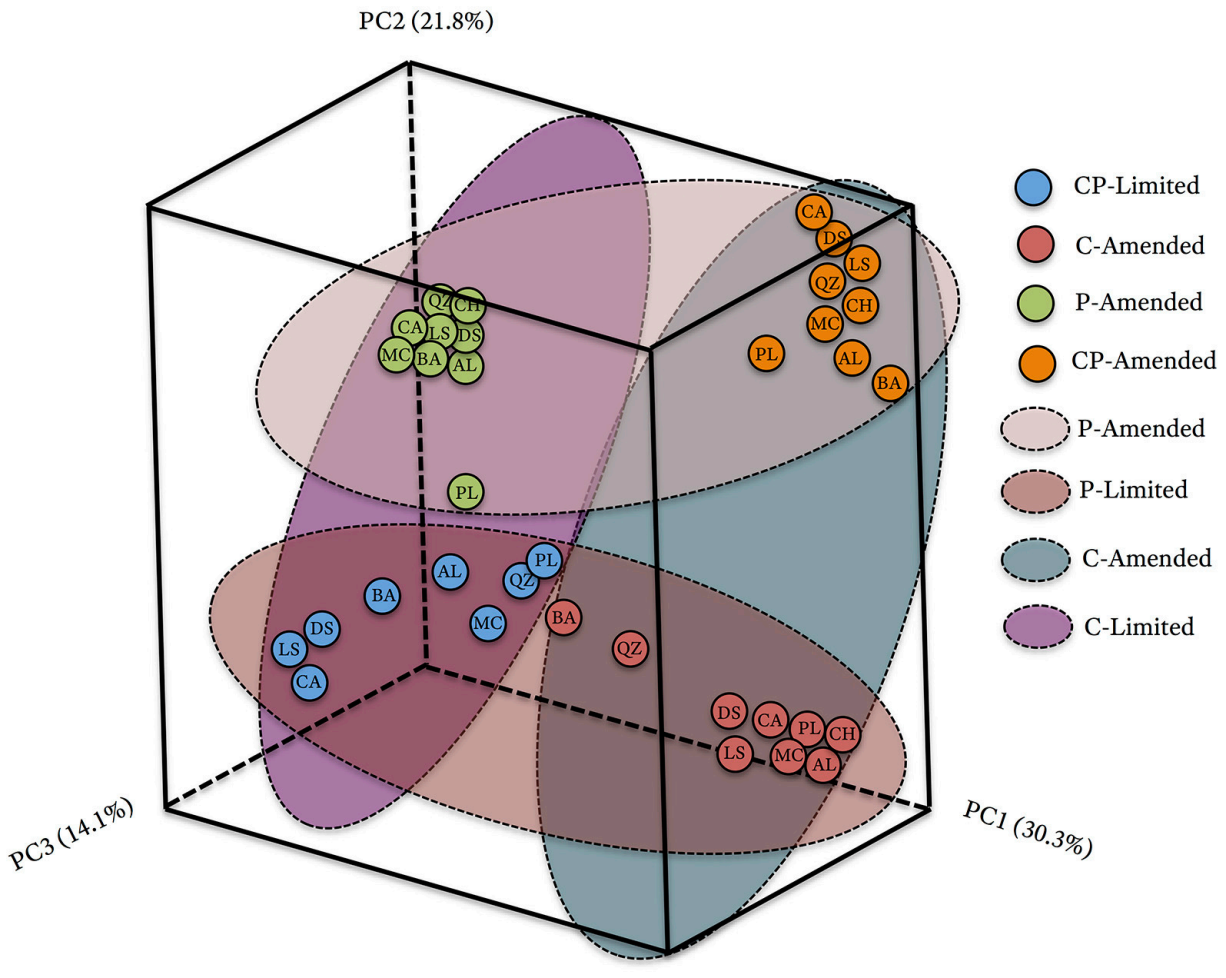
**Principal coordinate analysis (PCoA) plot based on the relative abundances and phylogenetic diversity of 16S rRNA gene sequences using a UniFrac weighted distance matrix, colored according to reactor treatment conditions and labeled according to solid substrate type; blue, CP-Limited; green, P-Amended; orange, CP-Amended; red, C-Amended**. Percentage of the diversity distribution explained by each axis is indicated in the figure. The colored ellipses encircle variations in reactor treatment conditions.

The bacterial communities between treatments formed phylogenetically distinct clusters in ordination space (Figure 2). UniFrac (phylogenetic) differences between treatment communities were significantly distinct from each other (PERMANOVA *P* < 0.001, *R*^2^ = 45.6%,) with overall similarities between 40.6 and 67.5% (Figure 2, Table 3). Each of the treatment variables (carbon, phosphate, pH) contributed significantly (*P* < 0.001) to phylogenetic differences between reactors (Table 3). Phosphate was the most important treatment controlling variable (*R*^2^ = 23.2%), but carbon (*R*^2^ = 16.5%), and media pH buffering (*R*^2^ = 20.1%) were also significant (*P* < 0.001; Table 3).

**Table 3:** **Effects of treatment conditions and surface type on bacterial β-diversity**.

**Surface controlling variables^a^**	**CP-Limited**			**C-Amended**			**P-Amended**			**CP-Amended**		
	***F***	***P***	**(*R*^2^)**	***F***	***P***	**(*R*^2^)**	***F***	***P***	**(*R*^2^)**	***F***	***P***	**(*R*^2^)**
Buffering capacity	**2.3**	**0.036**	**12.3**	0.1	0.929	Neg	0.8	0.537	10.7	**29.3**	**0.012**	**73.4**
Mineral type	**3.1**	**0.009**	**69.8**	0.8	0.609	32.3	**10.2**	**0.035**	**85.9**	**12.5**	**0.003**	**88.3**
Mineral phosphate	1	0.344	8.8	**3.1**	**0.034**	**30.9**	0.651	0.613	8.5	0.1	0.784	1.9
**Treatment controlling variables**^b^	***F***	***P***	**(*****R***^2^**)**									
Media carbon	**6.5**	<**0.001**	**16.5**									
Media phosphate	**9.9**	<**0.001**	**23.2**									
Media pH_in_	**2.4**	<**0.001**	**6.7**									
Media pH_out_	**8.3**	<**0.001**	**20.1**									
**Treatments**^c^	***t***	***P***_adjust_	**Ø**_sim_									
CP-Limited vs. C-Amended	**2.6**	**0.029**	**46.9**									
CP-Limited vs. P-Amended	**5.8**	<**0.001**	**55.8**									
CP-Limited vs. CP-Amended	**4.4**	<**0.001**	**40.6**									
C-Amended vs. P-Amended	**4.1**	<**0.001**	**55.1**									
C-Amended vs. CP-Amended	**6.3**	<**0.001**	**48.1**									
P-Amended vs. CP-Amended	**8.6**	<**0.001**	**67.5**									

a Effects of surface type and

b treatment conditions as assessed by multivariate permutational analysis of variance (PERMANOVA). Surface factors are buffering capacity (high vs. low based on whether surface is a carbonate or non-carbonate), mineral type (carbonate, silicate, aluminosilicate, planktonic), and mineral phosphate (high vs. low). Treatment correlation factors are carbon amendment (yes vs. no), phosphate amendment (yes vs. no), media pH_in_ (high vs. low), and media pH_out_ (high vs. Low). Values represent the pseudo-F ratio (F), the permutation-based level of significance (P), and the “adonis” (R^2^). Values at P < 0.05 are shown in bold. Negative variance components (Neg) can result from underestimations of small or zero variances.

c *Pairwise comparisons between treatments. Values represent the univariate t-statistic (t) and the between treatment UniFrac (phylogenetic) similarity (Ø_sim_). The permutation-based level of significance was adjusted for multiple comparisons using the Benjamini-Hochberg procedure (P_adjust_). Values at P_adjust_ < 0.05 are shown in bold*.

**Figure 3:**
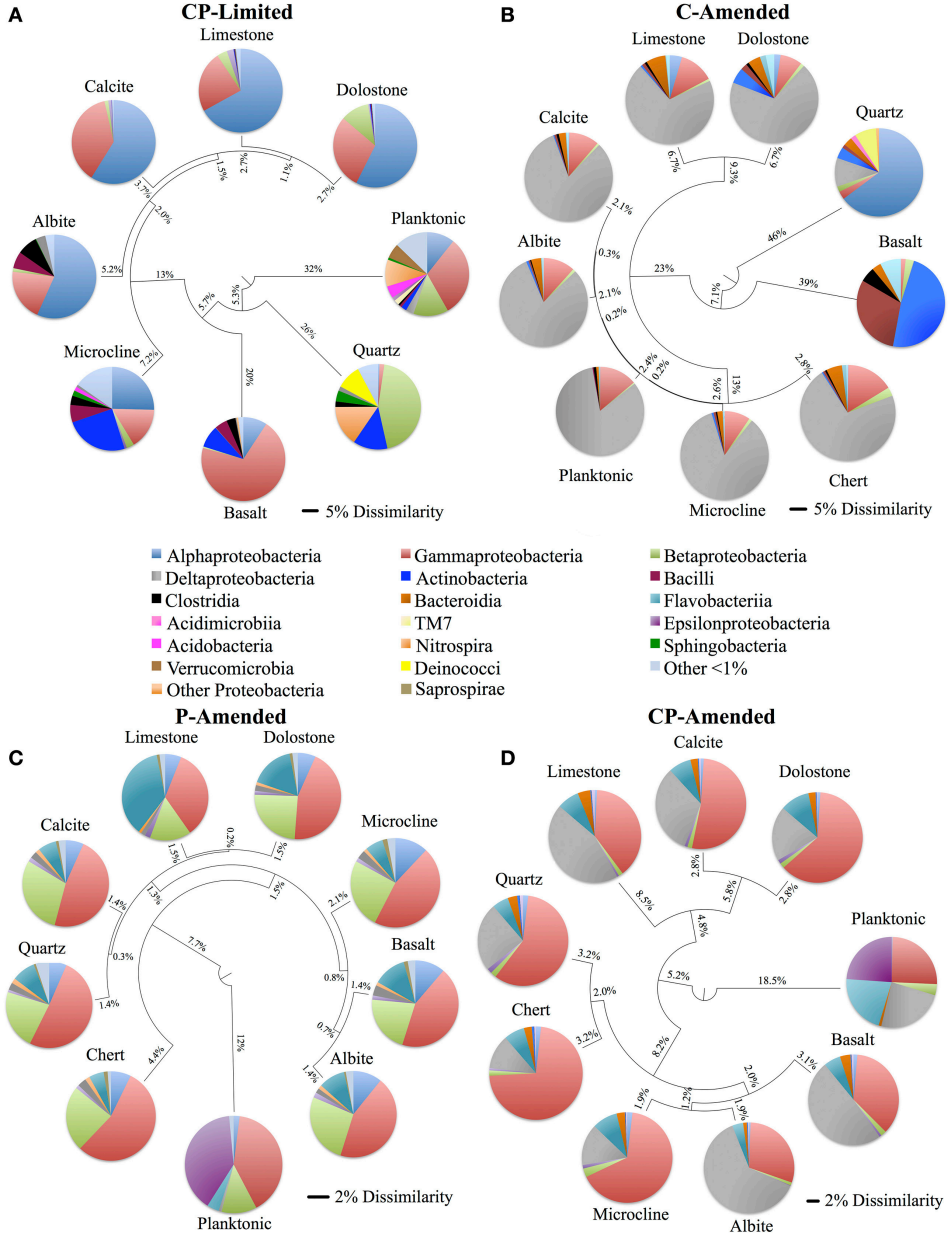
**Unweighted pair group method with arithmetic mean (UPGMA) trees constructed using weighted UniFrac (phylogenetic) distance matrix constructed from 16S rRNA sequences clustered at 97% similarity for each of the four reactor treatments (CP-Limited, P-Amended, C-Amended, CP-Amended)**. The trees display the phylogenetic overlap in bacterial communities colonizing various solid surfaces within each reactor treatment. The key (center) contains the class level taxonomy associated with each mineral surface (see Supplementary Tables 3–6 for proportional abundances). The scale bars for the CP-Limited and C-Amended treatments represents 0.05 (5%) dissimilarity in 16S rRNA sequences isolated from each surface and the scale bars for the P-Amended and CP-Amended treatments 0.02 (2%) dissimilarity. Note that although the taxonomy of the CP-Limited treatment is the same as Jones and Bennett (2014), the UPGMA tree here is based on UniFrac distances, while the tree in Jones and Bennett (2014; Figure 4) used the OTU based Sorenson similarity index.

Despite the effects of treatment, PERMANOVA revealed significant effects (*P* < 0.05) of surface type (carbonate, silicate, aluminosilicate, planktonic) on diversity within each reactor (Table 3). Trees constructed from UPGMA clustering of the UniFrac distance matrices serve to visualize differences in communities between mineral types (Figure 3). We applied these phylogenetic and statistical analysis to the oligotrophic treatment (CP-Limited) data from Jones and Bennett (2014). Here we confirmed that surface type (*R*^2^ = 69.8%, *P* = 0.009) and (*R*^2^ = 12.3%, *P* = 0.036) accounted for a majority of the variability in bacterial communities, while mineral phosphate (significant for growth) had no significant effect on community variability (Table 3). In both P-Amended reactors, surface type controlled >85% (*P* < 0.05) of the variance in phylogenetic diversity (*P* < 0.05) despite an overall decrease in phylogenetic variability between surface communities (Figure 3, Table 3). For the C-Amended treatment, mineral phosphate is the only statistically significant controlling variable (*R*^2^ = 30.9%, *P* = 0.034), but some clustering by surface type is apparent (Figure 3B). The C-Amended treatment had a planktonic community that was closely related to that of the surface communities (Figure 3). It should be noted that removal of the planktonic sample from PERMANOVA analysis makes surface type statistically significant (Table 3, Figure 3). Simply put, bacterial communities on similar surface types were statistically more phylogenetically similar for a given treatment (Figure 3).

Additionally, all of the treatment variables contributed significantly (*P* < 0.01, for all treatments) to the overall Shannon diversity of each treatment (Table 4). The Shannon diversity was significantly higher in C-limited and P-amended treatments, but species richness and number of unique OTUs were both significantly lower in both P-limited treatments (*P* = 0.0001; Table 4). Within separate treatments, mineral-buffering capacity was correlated with higher species richness (*P* = 0.024) and Shannon diversity (*P* = 0.036) in the CP-Limited treatment. In the C-Amended treatment, Shannon diversity was significantly higher on high-P surfaces (*P* = 0.007, Table 4). Within both the P-Amended and CP-Amended treatments, differences in alpha-diversity values were statistically insignificant (Table 4).

**Table 4:** **α-diversity summary and significance of mineralogy and treatment on α-diversity**.

**Surface^a^**	**CP-Limited**		**C-Amended**		**P-Amended**		**CP-Amended**	
	**Species richness (S)**	**Shannon diversity (H')**	**Species richness (S)**	**Shannon diversity (H')**	**Species richness (S)**	**Shannon diversity (H')**	**Species richness (S)**	**Shannon diversity (H')**
Calcite	362	6.38	195	2.41	332	6.49	534	6.53
Limestone	289	6.20	149	3.74	326	6.57	561	5.28
Dolostone	318	6.10	129	3.69	337	6.69	435	4.14
Basalt	71	3.04	115	5.21	332	6.55	323	4.27
Quartz	55	3.67	54	3.80	332	6.68	548	5.23
Albite	57	4.96	210	2.57	329	6.65	547	5.78
Microcline	116	6.33	190	2.27	301	6.43	381	5.00
Chert	0	0	143	2.93	300	6.18	542	5.55
Planktonic	133	5.79	120	1.90	63	2.24	424	5.65
Whole Reactor	349	6.63	386	3.56	459	6.54	757	5.63
Mean ± s.e.	175 ± 127	5.31 ± 1.30	145 ± 49	3.17 ± 1.04	295 ± 88	6.05 ± 1.44	477 ± 88	5.27 ± 0.74
**Surface correlation factors**^b^	***F*** **(*****P*****)**	***F*** **(*****P*****)**	***F*** **(*****P*****)**	***F*** **(*****P*****)**	***F*** **(*****P*****)**	***F*** **(*****P*****)**	***F*** **(*****P*****)**	***F*** **(*****P*****)**
Buffering capacity	**7.6 (0.024)**	**3.2 (0.036)**	1.1 (0.328)	0.8 (0.461)	0.9 (0.461)	0.7 (0.566)	0.8 (0.399)	0.1 (0.892)
Mineral type	5.6 (0.556)	1.1 (0.624)	3.9 (0.606)	2.2 (0.420)	0.4 (0.996)	0.3 (0.991)	0.6 (0.996)	0.2 (0.884)
Mineral phosphate	0.7 (0.461)	0.18 (0.834)	0.2 (0.863)	**4.3 (0.007)**	0.9 (0.602)	1.0 (0.428)	1.3 (0.201)	1.7 (0.061)
**Treatment correlation factors**^b^	**Species richness** ***F*** **(*****P*****)**						**Shannon diversity** ***F*** **(*****P*****)**	
Carbon Amendment	1.1 (0.278)						**3.1 (0.006)**	
Phosphate Amendment	**6.9 (0.0001)**						**3.2 (0.004)**	
Media pHin	**2.8 (0.011)**						**3.2 (0.003)**	
Media pHout	1.1 (0.301)						0.3 (0.758)	
**Treatments**^c^	**Species richness** ***F*** **(*****P*****)**						**Shannon diversity** ***F*** **(*****P*****)**	
CP-Limited vs. C-Amended	0.4 (0.892)						**3.2 (0.048)**	
P-Amended vs. CP-Amended	0.1 (0.887)						1.7 (0.656)	
P-Amended vs. C-Amended	**5.1 (0.002)**						**4.2 (0.008)**	
P-Amended vs. CP-Limited	**3.6 (0.042)**						1.8 (0.709)	
CP-Limited vs. CP-Amended	**4.5 (0.005)**						0.7 (0.997)	
C-Amended vs. CP-Amended	**7.2 (0.001)**						**3.7 (0.006)**	

a *Measures of species richness (S) and Shannon Diversity (H') for each surface and each treatment. Values are based on rarefied data sets for comparison across treatments*.

b *Impact of surface factors and treatments assessed by PERMANOVA. Surface factors are buffering capacity (high vs. low based on surface as a carbonate or non-carbonate), mineral type (carbonate, silicate, aluminosilicate, planktonic), and mineral phosphate (high vs. low). Treatment factors are carbon amendment (yes vs. no), phosphate amendment (yes vs. no), media pH_in_ (high vs. low), and media pH_out_ (high vs. low). Values are pseudo-F ratio (F) and the level of significance (P). P < 0.05 indicate a significant difference in a factor (shown in bold)*.

c *Pairwise comparisons by treatment use PERMANOVA*.

### Taxonomic composition and condition sensitive taxa

**Figure 4:**
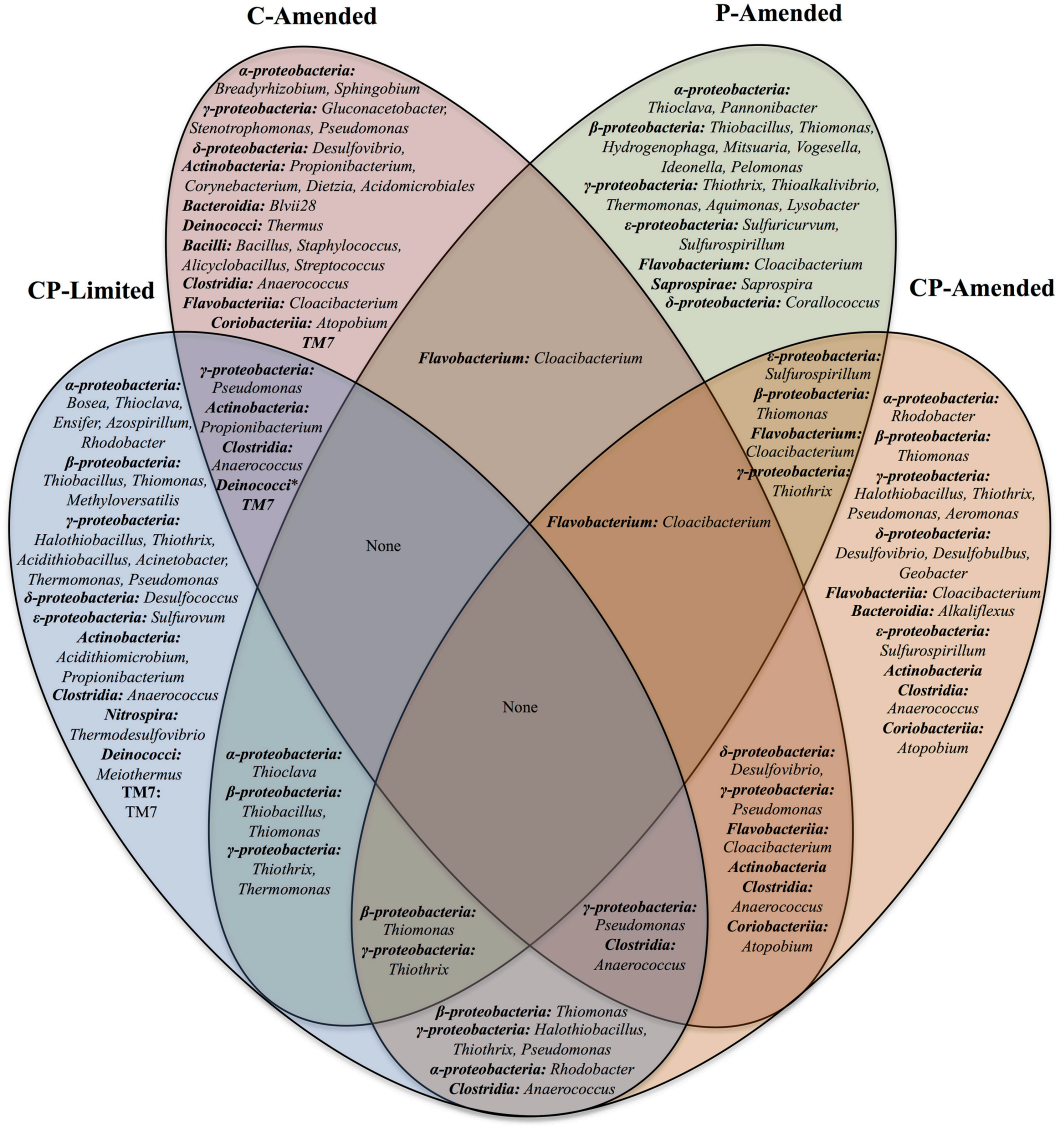
**Venn diagram of the shared bacterial genera found in the four reactor treatments (CP-Limited, P-Amended, C-Amended, CP-Amended)**. Community membership overlaps between the reactors are indicated by overlaps in the diagram.

Upon taxonomy assignment, OTUs were affiliated with 280 classified bacterial genera. Of those, 73 genera had relative abundances of more than 1%. These microbial communities include 20 bacterial classes: *Alphaproteobacteria, Betaproteobacteria, Deltaproteobacteria, Gammaproteobacteria, Epsilonproteobacteria, Acidobacteria, Acidomicrobiia, Actinobacteria, Bacilli, Bacteroidia, Clostridia, Coriobacteriia, Cytophagia, Deinococci, Flavobacteria, Nitrospira, Opitutae, Saprospirae, Sphingobacteria*, and *TM7* (Supplementary Tables 3–6). *Proteobacteria* was the most abundant Phylum across treatments. At the genus level there is very little overlap between overall experiment communities and no taxa common to all 4 experimental treatments (Figure 4).

In the CP-Limited treatment distinct lineages were constrained by mineralogy and mineral buffering capacity (Jones and Bennett, 2014). Briefly, neutrophilic autotrophic sulfur-oxidizing members of the lineages *Gammaproteobacteria, Betaproteobacteria*, and *Alphaproteobacteria*, were detected on highly buffering carbonates, Gram-positive *Actinobacter, Bacilli*, and *Clostridia* were detected almost exclusively on silicates, and representatives of acidophilic sulfur-oxidizing genera *Acidithiomicrobium, Thermodesulfovibrio*, and *Acidithiobacillus* detected exclusively on non-buffering quartz (Supplementary Table 3; Jones and Bennett, 2014). Similarly, samples from the P-Amended and CP-Amended treatments were dominated by autotrophic sulfur-oxidizing members of these lineages. However, these representatives were distributed on all surfaces within these treatments (Supplementary Table 3). P-Amended treatment samples hosted *Gammaproteobacteria* (34.2–54.9%), *Betaproteobacteria* (16.0–32.1%), and *Alphaproteobacteria* (6.3–12.6%); as representatives of the genera *Thiothrix, Thiomonas, Thiobacillus*, and *Thioclava* (Supplementary Table 3). In the CP-Amended treatment samples, sulfur-oxidizing *Gammaproteobacteria* (*Halothiobacillus* (9.6–49.5%) were most prominent, but lesser quantities of *Thiothrix* (<3.0%), and *Thiomonas* (<1.0%) were detected (Supplementary Table 4). Gram-positive bacteria were nearly absent (<1%) on all surfaces in both P-amended treatments, and not detectable in either planktonic sample (Supplementary Tables 4, 6). However, Gram-positives were detected on all surfaces in the C-Amended reactor as members of the lineages *Actinobacteria, Bacilli, Clostridia*, and *TM7* (Supplementary Table 5). *Actinobacteria* and *Bacilli* were particularly abundant in the Basalt sample. Additionally, representatives of the only genus detected in 3 treatments (*Cloacibacterium*) were abundant on all surfaces within the P-Amended (7.1–38.6%) and CP-Amended treatments (3.7–21.4%), but less abundant in the C-Amended samples (Supplementary Tables 4–6; Figure 4).

Although absent from both C-limited treatments, representatives of the heterotrophic sulfur-reducing lineage *Deltaproteobacteria* (*Desulfovibrio*) were present in both the C-Amended and CP-Amended treatments (Figure 4). Abundant *Desulfovibrio* was distributed amongst all samples within the CP-Amended treatment (11.6–61.4%), but not detected in the C-Amended basalt sample although it is found in higher overall abundance (69.1–84.5%) in the remaining C-Amended samples (Supplementary Tables 4, 5). The CP-Amended treatment was the only treatment to host significant quantities of both sulfur-oxiders (i.e., *Halothiobacillus*) and sulfur-reducers (i.e., *Desulfovibrio*; Supplementary Table 4).

A majority of the microbes in the inoculant came from two dominant classes (*Epsilonproteobacteria* and *Gammaproteobacteria*) representing ~97.1% of the total sequences (Supplementary Table 3; Jones and Bennett, 2014). Despite the dominance of *Epsilonproteobacteria* in the inoculant, *Epsilonproteobacteria* were all but absent in surface samples from every treatment, but present in appreciable amounts in the planktonic samples obtained from the P-Amended (39.5% as *Sulfuricurvum*) and CP-Amended (23.9% as *Sulfurospirillum*) treatments (Figure 3, Supplementary Tables 3–6). The CP-Limited planktonic sample is both phylogenetically and taxonomically distinct from any of the attached communities (Figure 3A). The primary differences taxonomically are the significant abundance of *Acidithiobacillus, Chloroacidobacterium, Acidisphaera, Thiobacillus*, and *Thermodesulfovibrio*).

## Discussion

The subsurface environment is a complex blend of rocks and minerals that are generally not considered to play an active role in microbial colonization. Using laboratory flow-through biofilm reactors, our study revealed that the media chemistry and the minerals present in the media significantly affect growth, diversity, and composition of a subsurface microbial community. Remarkably, 16S rRNA pyrosequencing revealed that microbial communities were mainly controlled by the media chemistry at the taxonomic level and by mineral type at the phylogenetic level. This study reveals significant evidence that mineral colonization is a non-random process controlled by both environmental and mineral conditions.

Mineral selectivity and local environmental geochemistry have been shown to impact the structure of attached microbial communities in soils, surface outcrops, and aquatic environments (Bennett et al., 2001; Gleeson et al., 2006; Carson et al., 2009; Hartmann et al., 2015; Uroz et al., 2015). However, it is currently unclear what motivates specific microbial communities to colonize specific surfaces. Here, we show that media pH, phosphate amendments, and carbon amendments significantly impacted the overall community taxonomy, but the phylogenetic distribution of that community among surfaces is ultimately correlated to surface type. Additionally, phosphate amendments, carbon amendments, and surface phosphate availability all significantly impacted biofilm accumulation. In fact, mineral phosphate concentration exerted significant control on biomass accumulation under all treatment conditions (Figure 1). Overall taxonomy and proportional abundance were significantly sensitive to variations in media and surface chemistry with consistent patterns emerging among specific guilds (SOB, SRB, Gram-positives, acidophiles).

### Keystone microorganisms

Looking at the total communities within each treatment, the main factor affecting taxonomy is the presence/absence of carbon amendments. C-Limited treatments were dominated by autotrophic SOB and C-Amended by heterotrophic SRB (Supplementary Tables 3–6). These taxonomic shifts in keystone microorganisms have diametrically opposing geochemical consequences due to differences in metabolic processes. Mineral selection is then a consequence of the reactivity of the surface to the metabolic byproducts of these keystone organisms and their environmental tolerances.

Locally, SOB benefit from attachment to carbonates; buffering the acidity generated by sulfur-oxidation to sulfate:

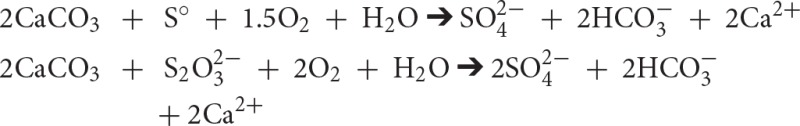



Previously, we demonstrated a preference for carbonates by the genera *Thiothrix, Thioclava, Halothiobacillus, Thiobacillus, Bosea, Thiomonas*, and *Sulfurovum* (Jones and Bennett, 2014). Aggressive dissolution of carbonates (Calcite, Madison Limestone, and Madison Dolostone) was confirmed by SEM analysis (Jones and Bennett, 2014).

If SOB-carbonate surface selection was linked to a need for pH buffering, then this preference would be less pronounced in a media buffered environment. Indeed, within the P-Amended treatment, media buffering reduced the dependence of neutrophilic SOB on mineral buffering of metabolically generated acidity (Supplementary Table 6). In the P-Amended treatment, potential SOB were identified ubiquitously on every surface, represented by the genera *Thiothrix, Thioalkalivibrio, Thiomonas*, and *Thiobacillus* (Supplementary Table 6). Obligately alkaliphilic *Thioalkalivibrio* are found exclusively within this treatment (Supplementary Tables 3–6; Sorokin et al., 2006). UniFrac analysis showed that all communities were very similar (>91% similar; Figure 3C). Despite this high degree of similarity, 85.9% (*P* = 0.035) of the phylogenetic variability in the P-Amended treatment was controlled by overall variations in mineral chemistry. Regardless, both of these treatment communities were composed of chemoautotrophic microorganisms, as the treatment media was carbon limited.

The addition of a carbon source (in the form of acetate, lactate, and formate) to the media promotes chemoheterotrophic growth. Both of the C-Amended treatments have significant quantities of *Deltaproteobacteria* represented by members of the heterotrophic sulfur-reducing genus *Desulfovibrio* (Supplementary Tables 4, 5). *Desulfovibrio* is a motile, vibrio-shaped, heterotrophic, sulfur-reducing bacteria capable of growth on a variety of sulfur substrates (as the terminal electron acceptor) as well as lactate, pyruvate, acetate, propionate, and butyrate (as electron donor and carbon source; Liu and Peck, 1981; Cypionka, 2000).

Metabolic sulfate reduction by SRB generally causes an increase in pH (Lyons et al., 1984; Walter et al., 1993; Van Lith et al., 2003; Dupraz et al., 2009). The geochemical consequences of sulfur-reduction by *Desulfovibrio* are localized consumption of acidity by paired acetate (shown below), lactate, or formate oxidation with reduction of inorganic sulfur compounds.

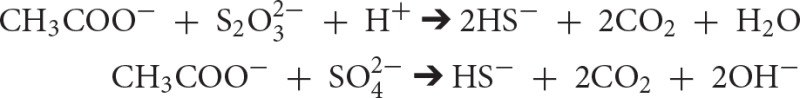



Also, in aerobic environments, several species of *Desulfovibrio* have been shown to pair sulfur-reduction with O_2_ reduction to H_2_O (Cypionka, 2000).

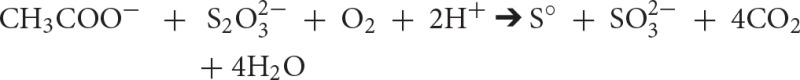



Within the C-Amended treatments, the metabolism of SRB allows more favorable conditions to be established on feldspars (albite & microcline) where consumption of local acidity decreases the mobility of potentially toxic mineral-bound aluminum (Rogers and Bennett, 2004). The relatively high pHs of both P-Amended medias reduces the dependence of neutrophilic, but acid-producing SOB for highly-buffering carbonates as media buffering facilitated acid consumption. Consequently, neutrophilic SOB colonized all surfaces at relatively high proportional abundances (Supplementary Table 6). Additionally, this decreases the overall species richness (alpha-diversity) as well as increases the amount of shared species (beta-diversity) between the surfaces (Figure 3B, Table 4). Overall, the dominance of these organisms was statistically correlated with lower Shannon diversity and β-diversity with less phylogenetic diversity between surfaces within C-Amended treatments (Table 4, Figures 3B,D). Decreased species richness has been documented to be a function of increasing carbon concentration (Larson and Passy, 2013). However, to our knowledge, a decrease in species diversity in attached communities as a function of carbon limitation across multiple surfaces is previously undocumented.

### Mineral specific accessory microorganisms

For the oligotrophic (CP-Limited) treatment taxonomy is clearly distinct for every mineral type (Figure 3A). Generally, for the other treatments, taxonomy is nearly identical on every surface. We found that surface type controlled a significant proportion of the variance in phylogenetic β-diversity for each treatment (Table 3). Geochemical conditioning of the near surface habitat by the keystone microorganisms is likely best suited for specific suites of accessory microorganisms.

Gram-positive bacteria are more attracted to silicate surfaces in low pH environments (Gordienko and Kurdish, 2007; Winsley et al., 2014). In both of the low-pH (and low-P) treatments (CP-Limited and C-Amended) there is a clear bias for Gram-positive bacteria on silicate surfaces (Supplementary Tables 3, 5). Furthermore, Gram-positives are negligible on all surfaces in the high-pH/C-limited treatment (P-amended). This suggests that pH and C may be the controlling factors on Gram-positive membership in microbial biofilm communities and additionally explains the lack of Gram-positive organisms within either of the treatments (P-Amended and CP-Amended) where a high pH was sustained (Supplementary Tables 4, 6). In the C-Amended treatment, Gram-positive *Actinobacteria, Bacilli*, and *Clostridia* composed a large proportion (86.8%) of the microbial community associated with basalt (Supplementary Table 5). Recent studies demonstrated that the ability of *Actinobacteria* and other Gram-positive microorganisms to weather basaltic materials in order to access mineral bound nutrients is increased significantly when provided a carbon source (Cockell et al., 2013). In the C-Amended treatment, *Alphaproteobacteria* with glycosphingolipids (GSLs) (*Sphingobium, Blastomonas*, and *Novosphingobium*) show an affinity for the quartz surface (Supplementary Table 5). Members of this lineage are known for strong silicate surface adhesion in oligotrophic and extreme environments and well-modulated cellular pH (Eguchi et al., 1996; Laskin and White, 1999; Yamaguchi and Kasamo, 2002; Sun et al., 2013; Varela et al., 2014). The Quartz surface was the ideal habitat for these and acidophilic microorganisms within the C-Amended treatment due to their unique ability to tolerate low-pH environments without having to compete with the dominant keystone heterotrophic SRB within the reactor.

Additionally, the relatively basic pH of the P-Amended treatment provided an environmental advantage for potentially alkaliphilic microorganisms, constituting a proportional abundance of 13.3–28.1% on all surfaces (Supplementary Table 6). The most abundant of these organisms were members of the class β*-proteobacteria*. Of this lineage, *Hydrogenophaga* was the most abundant on all surfaces. Hydrogenophaga is a well-known aerobic, hydrogen-oxidizing microorganism commonly associated with subsurface serpentinization processes. Although many members of this genus were thought to be obligately neutrophilic, recently many members of this lineage have been isolated from and shown to thrive in high-alkalinity environments (Willems et al., 1989; Roadcap et al., 2005; Suzuki et al., 2013).

### Biomass accumulation

Nutrient limitations can stimulate biomass growth and production of EPS (Ellwood et al., 1982; Matin et al., 1989; Wrangstadh et al., 1990; Zisu and Shah, 2003; Eboigbodin et al., 2007). Here, significantly higher biofilm was positively correlated with carbon amendments (*P* < 0.04) independent of media phosphate, mineral phosphate, and media pH (Figure 1, Supplementary Table 1). Both of the C-Amended treatments accumulated ~2X the total biomass of their respective P-Amended counterparts (CP-Limited 50.5 mg·cm^−2^ vs. C-Amended 110.1 mg·cm^−2^, and P-Amended 12.8 mg·cm^−2^ vs. CP-Amended 25.4 mg·cm^−2^; Supplementary Table 1). Carbon limitations in the C-Limited treatments decreased the metabolic efficacy of heterotrophic populations (Matin et al., 1989). Previous studies have also demonstrated an increase in biofilm formation by heterotrophic bacteria in response to nutrient limitations (Matin et al., 1989; Wrangstadh et al., 1990). It should be noted that this dramatic increase in biofilm concentration is complex as it is likely tied to dynamic biochemical and biophysical interactions of specific heterotrophic taxa with specific surfaces and treatments (Supplementary Tables 4, 6).

P-Amended treatments had significantly lower biofilm biomass (*P* < 0.002), but high-phosphate surfaces (limestone, dolostone, basalt) had significantly higher biomass (2–60X; Figure 1). Previously, we found that the primary control on total biomass accumulation was the concentration of mineral bound nutrients, particularly in the form of phosphate (Figure 1; Jones and Bennett, 2014). Several studies have reported that microorganisms exhibit active adhesion/detachment processes that may be a response to local nutrient availability (Dawson et al., 1981; Kjelleberg and Hermansson, 1984; Van Loosdrecht et al., 1990; Wrangstadh et al., 1990; Marshall, 1996; Araújo et al., 2010). In particular, these investigators have noted that starvation or nutrient availability can stimulate a change in the partitioning of a microbial community between the solid and aqueous phases (Ginn et al., 2002). During starvation bacteria show increased levels of adhesion by increasing production of EPS, allowing them to take advantage of organic and inorganic compounds that accumulate at solid-liquid interfaces (Dawson et al., 1981).

These previous investigations speculated that such tactics might be particularly important in oligotrophic waters where bacteria are exposed to conditions of extreme nutrient limitation. We observed that bacteria exploit these tactics in a multitude of nutrient and environmental conditions. In P-Amended treatments the magnitude of actual variation (standard deviation) in total biomass between high-P and low-P surfaces was much lower (Figure 1, Supplementary Table 1). It is easy to intuit that the availability of media phosphate reduces the reliance on surface-bound phosphate for survival, but these surfaces are still favored by non-motile bacteria.

### Planktonic vs. attached communities

In natural aquifers, microbial populations differentiate between planktonic and surface attached communities (Lehman et al., 2001). Here, for every treatment, the planktonic communities and inoculants were taxonomically and phylogenetically distinct from attached communities (Figure 3, Supplementary Tables 3–6). However, in the P-Amended media these distinctions were less apparent. Unlike oligotrophic media (P-Amended) there is a less significant advantage to attachment. Previous investigations have used this explanation for distinctions between planktonic and attached communities (Hazen et al., 1991; Zhou et al., 2012).

Additionally, this might explain the high degree of phylogenetic similarity (67.5%) between the two P-Amended treatments. Both P-Amended treatments had high proportions of *Epsilonproteobacteria* found exclusively in these samples (Figure 3, Supplementary Tables 3–6). These lineages were composed entirely of members of the genera *Sulfuricurvum* and *Sulfurospirillum*, within the P-Amended and CP-Amended treatments, respectively. *Sulfuricurvum* is a motile, chemolithoautotrophic SOB which grows best at near neutral pHs (Kodama and Watanabe, 2004). *Sulfurospirillum* is a motile, chemoheterotrophic, SRB which also grows best at near neutral pHs and is capable of using acetate as a carbon source and acetate and formate as electron donors (Kodama et al., 2007). These common treatment media conditions of phosphate availability and neutral to high pH, combined with the motility of these organisms, favor planktonic existence.

Despite phylogenetic similarities between treatments, there are no taxa common to all 4 experimental treatments (Table 3, Figure 4). This result was unexpected, but serves to underscore the fundamental role that environment plays in community membership. In truly oligotrophic and unfavorable conditions (CP-Limited), specific microorganisms are highly dependent on specific minerals for survival. This leads to taxonomically and phylogenetically distinct communities segregated by mineral type (Figure 3A). Variability in environmental conditions (i.e., pH, C, P) influences viability of specific guilds, which facilitates specific mineral selective strategies. Each experimental treatment and surface type represented a unique habitat that encouraged growth of both major (keystone) and minor (accessory) populations and revealed the presence of hundreds of additional OTUs that were below detection in the original inoculant.

In conclusion, we reported a statistically significant link between phylogenetic diversity of microbial communities and specific natural surface types under a variety of geochemical conditions. This suggests that phylogenetically similar microorganisms are significantly more likely to have similar surface habitat requirements. In resource stressed and harsh environments, minerals act as environmental filters providing specific microhabitats for metabolically similar microorganisms. Successful growth and succession is a function of the capacity of a microorganism, or community, to facilitate, tolerate, or adapt to microenvironmental cultivation and modification of the geochemical conditions at the microbe-mineral-aqueous interface. Environmental pressures such as pH, carbon, and/or phosphate variability will determine the extent of taxonomic disparity between mineral microniches, but surface type ultimately controls the phylogenetic diversity between these microenvironments. Due to their unique and variable chemical compositions, rocks and minerals should be considered as ecosystems that are primarily colonized by uniquely adapted microbes. Further investigation is required to determine if these phylogenetic similarities are the result of ecological shifts caused by environmental stresses imposed by localized surface geochemistry or latent adaptations by specific keystone and accessory organisms.

## Author contributions

AJ conceived and designed the experiments. AJ acquired and processed the data. AJ and PB interpreted the data. AJ drafted the original work as part of his dissertation at The University of Texas at Austin entitled: Mineralogical Controls on Microbial Community Structure and Biogeochemical Processes in Subsurface Environments. AJ and PB revised this work for important intellectual contend and agreed on a final version to be submitted for publication. Therefore, AJ and PB both agree to be accountable for all aspects of this work in ensuring that questions related to the accuracy or integrity of any part of the work are appropriately investigated and resolved.

### Conflict of interest statement

The authors declare that the research was conducted in the absence of any commercial or financial relationships that could be construed as a potential conflict of interest.
