# Supplementary material for: Mineral Ecology: Surface Specific Colonization and Geochemical Drivers of Biofilm Accumulation, Composition, and Phylogeny
Source: Front Microbiol. 2017 Mar 28;8:491. doi: 10.3389/fmicb.2017.00491 (PMC5368280; doi:10.3389/fmicb.2017.00491)
Supplement: Supplementary file 1 [file Table1.PDF]

Surface	Dry Mass of Biofilm (mg/cm^2)			
	CP-Limited[c]	C-Amended	P-Amended	CP-Amended
Calcite	2.4 ± 0.3	4.5 ± 0.4	0.4 ± 0.2	1.3 ± 0.3
Limestone	18.9 ± 2.3	40.3 ± 2.4	5.6 ± 1.4	9.6 ± 2.1
Dolostone	18.8 ± 4.1	36.2 ± 3.7	4.8 ± 1.2	8.7 ± 1.0
Microcline	0.5 ± 0.4	4.2 ± 0.8	0.1 ± 0.1	0.8 ± 0.3
Albite	1.2 ± 0.3	4.8 ± 0.8	0.2 ± 0.2	0.6 ± 0.1
Chert	0.5 ± 0.1	5.2 ± 2.3	0.1 ± 0.1	0.4 ± 0.1
Basalt	6.0 ± 1.4	9.1 ± 0.8	1.4 ± 1.1	3.2 ± 0.6
Quartz	2.2 ± 0.7	5.8 ± 1.5	0.2 ± 0.1	0.8 ± 0.1
Whole Reactor[a]	50.5	110.1	12.8	25.4
Standard Deviation[b]	8.0	15.2	2.3	3.8

Supplementary Table 1: Dry mass of biofilm values and standard deviations. The standard deviations for all experiments are the result of triplicate experiments with dry biomass values as the mean.

[a]Sum of the mean values of dry biomass for each treatment as a whole reactor.

[b]Standard deviation in for dry biomasses on surfaces within each reactor treatment.

[c]Dry biomass of biofilm values for CP-Limited treatment from Jones and Bennett 2014.
