# Supplementary material for: Mineral Ecology: Surface Specific Colonization and Geochemical Drivers of Biofilm Accumulation, Composition, and Phylogeny
Source: Front Microbiol. 2017 Mar 28;8:491. doi: 10.3389/fmicb.2017.00491 (PMC5368280; doi:10.3389/fmicb.2017.00491)
Supplement: Supplementary file 2 [file Table2.PDF]

Treatment	Surface	SAC Accession Number	High-Quality Seqs.	Species Richness (S)	Shannon Diversity (H')	Evenness (E)	Simpson's Index (1-D)	Inverse Simpson (1/(1-D))	Good's Coverage
CP-Limited	Calcite	SAMN05181938	7829	362	6.38	1.08	0.03	35.31	99.7%
	Limestone	SAMN05181940	6095	289	6.20	1.09	0.03	39.63	99.4%
	Dolostone	SAMN05181939	7279	318	6.10	1.06	0.03	34.65	99.5%
	Basalt	SAMN05181937	1136	71	3.04	0.71	0.35	2.84	98.7%
	Quartz	SAMN05181943	2315	55	3.67	0.91	0.15	6.89	99.8%
	Albite	SAMN05181936	396	57	4.96	1.23	0.05	22.05	95.7%
	Microcline	SAMN05181941	546	116	6.33	1.33	0.02	55.25	95.1%
	Planktonic	SAMN05181942	1577	133	5.79	1.18	0.05	21.86	99.6%
	Whole Reactor		27173	349	6.63	0.98	0.02	56.85	99.9%
	Mean		3397	175	5.31	1.08	0.09	27.31	98.4%
	Standard Dev		3133.3	126.9	1.30	0.19	0.12	17.42	1.9%
C-Amended	Calcite	SAMN05181929	10894	195	2.41	0.46	0.47	2.15	99.5%
	Limestone	SAMN05181932	5604	149	3.74	0.75	0.22	4.64	99.6%
	Dolostone	SAMN05181931	2792	129	3.69	0.76	0.22	4.57	99.2%
	Basalt	SAMN05181928	6555	115	5.21	1.10	0.07	13.80	99.8%
	Quartz	SAMN05181935	594	54	3.80	0.95	0.14	7.03	99.5%
	Albite	SAMN05181927	16531	210	2.57	0.48	0.43	2.32	99.8%
	Microcline	SAMN05181933	15484	190	2.27	0.43	0.48	2.08	99.8%
	Chert	SAMN05181930	3484	143	2.93	0.59	0.38	2.66	98.6%
	Planktonic	SAMN05181934	4445	120	1.90	0.40	0.54	1.86	99.0%
	Whole Reactor		66383	386	3.56	0.78	0.22	4.47	100.0%
	Mean		7376	145	3.17	0.66	0.33	4.57	99.4%
	Standard Dev		5662.3	48.5	1.04	0.25	0.17	3.86	0.4%
P-Amended	Calcite	SAMN05181955	4538	332	6.49	1.12	0.02	42.00	98.1%
	Limestone	SAMN05181958	4146	326	6.57	1.14	0.02	42.70	98.2%
	Dolostone	SAMN05181957	4302	337	6.69	1.15	0.02	49.14	98.1%
	Basalt	SAMN05181954	3997	332	6.55	1.13	0.02	43.20	97.8%
	Quartz	SAMN05181961	3945	332	6.68	1.15	0.02	45.02	98.2%
	Albite	SAMN05181953	4099	329	6.65	1.15	0.02	47.43	97.9%
	Microcline	SAMN05181959	2820	301	6.43	1.13	0.03	36.03	96.5%
	Chert	SAMN05181956	3668	300	6.18	1.08	0.04	27.78	97.5%
	Planktonic	SAMN05181960	6332	63	2.24	0.54	0.39	2.56	99.8%
	Whole Reactor		37847	459	6.54	0.97	0.03	33.74	99.9%
	Mean		4205	295	6.05	1.06	0.07	37.32	98.0%
	Standard Dev		933.8	87.9	1.44	0.20	0.12	14.53	0.9%
CP-Amended	Calcite	SAMN05181946	6589	534	6.53	1.04	0.04	25.15	97.7%
	Limestone	SAMN05181949	12751	561	5.28	0.83	0.10	10.43	98.9%
	Dolostone	SAMN05181948	12674	435	4.14	0.68	0.20	5.12	99.1%
	Basalt	SAMN05181945	5340	323	4.27	0.74	0.16	6.15	97.4%
	Quartz	SAMN05181952	11016	548	5.23	0.83	0.11	9.38	98.8%
	Albite	SAMN05181944	8230	547	5.78	0.92	0.07	14.73	98.0%
	Microcline	SAMN05181950	5077	381	5.00	0.84	0.11	9.09	96.9%
	Chert	SAMN05181947	8936	542	5.55	0.88	0.09	11.55	98.4%
	Planktonic	SAMN05181951	7957	424	5.65	0.93	0.06	17.25	98.2%
	Whole Reactor		78570	757	5.63	0.92	0.08	12.15	99.9%
	Mean		8730	477	5.27	0.86	0.10	12.10	98.1%
	Standard Dev		2899.6	88.0	0.74	0.11	0.05	6.20	0.7%

Supplementary Table 2. Bacterial α-diversity on each surface and for each whole reactor treatment. High-quality sequences are sequences used in diversity analyses. Species richness (S), Shannon Diversity (H'), Evenness (E), Simpson's diversity index (1-D), and Good's Coverage (%) for each surface and each reactor. Values based on rarefied data sets for sequences from surfaces within each reactor treatment and whole reactors for comparisons between treatments.
