# Supplementary material for: Mineral Ecology: Surface Specific Colonization and Geochemical Drivers of Biofilm Accumulation, Composition, and Phylogeny
Source: Front Microbiol. 2017 Mar 28;8:491. doi: 10.3389/fmicb.2017.00491 (PMC5368280; doi:10.3389/fmicb.2017.00491)
Supplement: Supplementary file 3 [file Table3.PDF]

Representative *Class*	Representative *Genus*	LKC Inoculant	Calcite	Madison Limestone	Madison Dolostone	Microcline	Albite	Quartz	Basalt	Planktonic
α-proteobacteria		**0.1**	**58.9**	**67.0**	**57.4**	**25.4**	**56.7**	**0.5**	**9.2**	**10.7**
	Ensifer	0	29.4	30.4	14.7	6.0	29.2	0	4.7	0
	Azospirillum	0	6.6	8.3	11.5	1.3	16.4	0	2.5	0
	Bosea	0	5.6	3.0	12.9	0.2	6.5	0	0	0
	Thioclava	0	6.0	3.1	7.1	0.1	0	0	0	0
	Defluvibacter	0	2.9	1.1	0.6	0.1	1.4	0	0.1	0.1
	Sphingopyxis	0	2.5	12.6	5.3	3.2	0	0	0.4	0
	Sphingomonas	0	0	0	0	3.2	0.2	0	0	0.1
	Acidisphaera	0	0	0	0	0	0	0.3	0	3.1
	Rhodobacter	0	0	0	0	0	0	0	0	1.5
γ-proteobacteria		**34.7**	**37.6**	**23.7**	**28.8**	**16.0**	**20.1**	**1.8**	**70.1**	**31.1**
	Acinetobacter	0	22.6	3.5	4.0	2.0	0	0.2	0.1	0
	Halothiobacillus	0	9.1	5.6	14.5	0.3	0	0	0	0
	Thermomonas	0	4.4	12.5	7.7	0.2	5.1	0	0	0
	Pseudomonas	0	0.8	0.4	0.4	4.5	10.4	0	0	0.2
	Thiothrix	34.7	0	0.2	0.2	0	0	0	65.5	0.2
	Stenotrophomonas	0	0	0	0.1	1.4	1.6	0	3.7	0
	Acidithiobacillus	0	0	0	0	0	0	1.2	0	25.9
	Nitrosococcus	0	0	0	0	0	0	0	0	2.2
β-proteobacteria		**0.2**	**1.5**	**4.0**	**11.0**	**2.9**	**1.4**	**44.1**	**0.6**	**13.5**
	Thiobacillus	0	0.3	0.5	0.7	0	0	1.2	0	5.9
	Methyloversatilis	0.1	0.2	0.9	0.9	0	0	26.4	0	0.4
	Zoogloea	0	0	0	0	0	0	3.5	0	2.5
	Thiomonas	0	1.0	2.5	9.1	0	0	0	0	0
ε-proteobacteria		**62.4**	**1.2**	**2.6**	**0.7**	**0.9**	**0**	**0**	**0**	**1.4**
	Sulfurovum	62.3	1.2	2.6	0.7	0.3	0	0	0	0
Actinobacteria		**0.1**	**0.1**	**0.3**	**0.4**	**24.6**	**0**	**13.1**	**8.6**	**2.1**
	Acidithiomicrobium	0	0	0	0	0	0	12.6	0	0.5
	Rubrobacter	0	0	0	0	3.9	0	0	0	0.1
	Propionibacterium	0	0.1	0.1	0.1	0.8	0	0.2	4.7	0
	Microthrix	0	0	0	0	0	0	0	0	1.5
Bacilli		**0**	**0.1**	**0.2**	**0.4**	**6.7**	**6.5**	**0**	**4.9**	**0.9**
	Lactobacillus	0	0.1	0.1	0.2	0.2	0.9	0	3.8	0.0
Clostridia	***Anaerococcus***	**0.9**	**0.1**	**0.1**	**0.1**	**3.6**	**7.9**	**2.1**	**3.5**	**0.7**
Sphingobacteria		**0**	**0**	**0**	**0**	**2.1**	**0.2**	**4.2**	**0**	**0.9**
Acidobacteria		**0**	**0**	**0**	**0**	**1.6**	**0**	**0**	**0**	**4.7**
	Chloroacidobacterium	**0**	**0**	**0**	**0**	**0**	**0**	**0**	**0**	**4.7**
δ-proteobacteria		**0.5**	**0.1**	**0.4**	**0.2**	**0.9**	**3.7**	**1.7**	**0.4**	**3.1**
	Desulfococcus	0	0	0.2	0	0	3.7	0	0	0
	Desulfomonile	0	0.1	0	0.1	0	0	0	0.4	0
Deinococci	***Meiothermus***	**0**	**0**	**0**	**0**	**0**	**0**	**9.4**	**0.7**	**0**
Nitrospira	***Thermodesulfovibrio***	**0**	**0**	**0**	**0**	**0**	**0**	**15.4**	**0.1**	**10.4**
Opitutae	***Opitutus***	**0**	**0**	**0**	**0**	**0**	**0**	**0**	**0**	**5.7**
TM7	***TM7***	**0**	**0**	**0**	**0**	**0**	**0**	**0**	**0**	**2.0**
***Class* <1% Abundance/Unclassified**		**0.5**	**0.5**	**1.8**	**1.1**	**15.3**	**3.5**	**7.8**	**2.0**	**12.8**
**Total Proportion SOB**		**97.0**	**23.3**	**17.4**	**45.2**	**0.9**	**6.5**	**15.0**	**65.5**	**32.5**
**Total Proportion Netrophilic SOB**		**97.0**	**23.3**	**17.4**	**45.2**	**0.9**	**6.5**	**1.2**	**65.5**	**6.1**
**Total Proportion Acidophilic SOB**		**0**	**0**	**0**	**0**	**1.6**	**0**	**13.8**	**0**	**26.4**
**Total Proportion Acidophilic**		**0**	**0**	**0**	**0.9**	**0**	**0**	**38.9**	**0.8**	**44.6**
**Total Proportion SRB**		**0**	**0.1**	**0.2**	**0.1**	**0**	**3.7**	**15.4**	**0.5**	**10.4**
**Total Proportion Gram Positive**		**1.0**	**0.3**	**0.6**	**0.9**	**34.9**	**14.4**	**15.2**	**17.0**	**5.7**

Supplementary Table 3. CP-Limited treatment samples as proportional abundance (%) of taxa of representative class (bold) and genera from 16S rRNA gene sequences, for each surface, and planktonic sample after 3-weeks within the CP-Limited reactor. Potential sulfur-oxidizing genera (SOB), sulfur-reducing genera (SRB), acidophilic genera, and gram-positive genera are highlighted (Adapted from Jones and Bennett 2014).
