# Supplementary material for: Mineral Ecology: Surface Specific Colonization and Geochemical Drivers of Biofilm Accumulation, Composition, and Phylogeny
Source: Front Microbiol. 2017 Mar 28;8:491. doi: 10.3389/fmicb.2017.00491 (PMC5368280; doi:10.3389/fmicb.2017.00491)
Supplement: Supplementary file 4 [file Table4.PDF]

Representative Class	Representative Genus	Calcite	Madison Limestone	Madison Dolostone	Microcline	Albite	Quartz	Basalt	Chert	Planktonic
γ-proteobacteria		**52.1**	**39.0**	**62.4**	**66.0**	**29.8**	**58.5**	**36.1**	**72.3**	**25.4**
	Pseudomonas	36.3	5.3	9.0	13.2	6.2	16.2	6.4	18.5	9.8
	Thiothrix	2.2	0.5	2.5	0.4	0.6	0.4	0.8	0.3	0.1
	Halothiobacillus	9.6	29.3	45.9	49.5	21.4	38.7	24.7	49.5	11.4
	Aeromonas	2.4	3.0	3.1	1.5	0.8	1.8	3.0	2.0	0.1
δ-proteobacteria		**32.3**	**44.2**	**19.5**	**14.9**	**62.3**	**24.5**	**48.8**	**11.9**	**24.0**
	Desulfovibrio	28.8	44.1	19.4	14.4	61.4	24.1	48.3	11.6	22.1
	Desulfobulbus	3.3	0.0	0.1	0.1	0.9	0.0	0.3	0.1	0.0
	Geobacter	0.1	0.1	0.0	0.4	0.0	0.4	0.2	0.2	1.8
Flavobacteriia		**8.1**	**7.8**	**10.6**	**9.1**	**3.9**	**5.8**	**6.0**	**7.1**	**21.4**
	Cloacibacterium	7.4	7.4	10.3	8.9	3.7	5.4	5.9	6.9	21.4
Bacteroidia		**2.7**	**4.4**	**2.6**	**3.0**	**1.3**	**3.4**	**3.7**	**2.7**	**1.0**
	Alkaliflexus	1.5	2.4	1.9	2.0	0.6	2.1	2.2	1.6	0.9
β-proteobacteria		**1.6**	**1.5**	**1.8**	**3.1**	**1.1**	**2.1**	**2.0**	**1.9**	**4.0**
	Thiomonas	0.0	0.1	0.5	0.4	0.2	0.5	0.4	0.2	0.0
	Comamonas	0.5	0.3	0.2	1.0	0.2	0.4	0.6	0.4	2.7
ε-proteobacteria		**1.1**	**0.7**	**1.4**	**1.2**	**0.2**	**1.8**	**0.8**	**0.8**	**23.9**
	Sulfurospirillum	1.0	0.7	1.4	1.2	0.2	1.7	0.8	0.8	23.9
α-proteobacteria		**1.0**	**0.8**	**1.1**	**1.9**	**0.6**	**1.9**	**1.4**	**1.8**	**0.3**
	Rhodobacter	0.8	0.6	0.9	1.4	0.3	1.5	0.8	1.2	0.1
Actinobacteria		**0.3**	**0.3**	**0.3**	**0.2**	**0.3**	**0.8**	**0.3**	**0.8**	**0.0**
Clostridia		**0.0**	**0.1**	**0.1**	**0.1**	**0.0**	**0.1**	**0.1**	**0.1**	**0.0**
	Clostridium	0.0	0.1	0.1	0.1	0.0	0.1	0.1	0.1	0.0
**Class <1% Abundance/ Unclassified**		**0.7**	**1.2**	**0.3**	**0.5**	**0.4**	**1.2**	**0.8**	**0.6**	**0.0**
**Total Proportion SOB**		11.9	29.9	48.9	50.2	22.2	39.6	25.8	50.0	11.5
**Total Proportion SRB**		33.1	44.9	20.9	15.7	62.4	25.8	49.4	12.5	46.0
**Total Proportion G+**		0.4	0.4	0.4	0.3	0.3	0.9	0.4	0.8	0.0

Supplementary Table 4. CP-Amended treatment samples as proportional abundance (%) of taxa of representative class (bold) and genera from 16S rRNA gene sequences for surfaces and planktonic sample after 3-weeks within the CP-Amended reactor. Potential sulfur-oxidizing genera (SOB), sulfur-reducing genera (SRB), acidophilic genera, and gram-positive genera are highlighted.
