# Supplementary material for: Mineral Ecology: Surface Specific Colonization and Geochemical Drivers of Biofilm Accumulation, Composition, and Phylogeny
Source: Front Microbiol. 2017 Mar 28;8:491. doi: 10.3389/fmicb.2017.00491 (PMC5368280; doi:10.3389/fmicb.2017.00491)
Supplement: Supplementary file 5 [file Table5.PDF]

Representative Class	Representative Genus	Calcite	Madison Limestone	Madison Dolostone	Microcline	Albite	Chert	Quartz	Basalt	Planktonic
δ-proteobacteria		**82.1**	**70.4**	**69.1**	**84.5**	**80.8**	**71.6**	**10.5**	**0.2**	**82.8**
	Desulfovibrio	82.1	70.4	69.1	84.5	80.8	71.6	10.1	0.2	82.8
α-proteobacteria		**0.2**	**4.5**	**2.3**	**0.1**	**0.0**	**0.2**	**64.8**	**0.0**	**0.1**
	Sphingobium	0.0	2.1	1.5	0.0	0.0	0.0	10.5	0.0	0.0
	Blastomonas	0.0	0.2	0.3	0.0	0.0	0.0	4.6	0.0	0.0
	Novosphingobium	0.0	0.1	0.0	0.0	0.0	0.0	3.0	0.0	0.0
	Bradyrhizobium	0.0	0.1	0.4	0.0	0.0	0.0	45.3	0.0	0.0
γ-proteobacteria		**11.1**	**12.7**	**8.1**	**9.4**	**11.6**	**16.0**	**3.0**	**1.8**	**13.8**
	Gluconacetobacter	5.2	8.4	6.4	3.1	5.3	4.0	1.3	0.0	6.6
	Stenotrophomonas	2.8	3.7	0.4	4.2	4.2	7.5	1.1	0.0	1.7
	Pseudomonas	1.4	0.2	0.7	1.2	0.9	2.0	0.0	0.0	4.5
β-proteobacteria		**0.8**	**0.9**	**1.3**	**1.1**	**1.1**	**3.0**	**2.0**	**2.9**	**0.5**
	Diaphorobacter	0.0	0.7	0.2	0.0	0.0	0.0	0.7	2.6	0.0
	Janthinobacterium	0.5	0.0	0.2	0.7	0.8	2.7	0.0	0.0	0.4
Actinobacteria		**0.4**	**0.7**	**5.8**	**0.7**	**0.9**	**0.5**	**4.2**	**48.0**	**0.0**
	Propionibacterium	0.0	0.2	1.6	0.0	0.0	0.0	2.6	35.7	0.0
	Acidomicrobiales	0.0	0.0	0.0	0.0	0.0	0.0	1.9	0.0	0.0
	Corynebacterium	0.0	0.0	2.4	0.0	0.0	0.0	0.7	2.5	0.0
	Dietzia	0.0	0.0	0.0	0.0	0.0	0.0	0.0	8.1	0.0
Bacilli		**0.8**	**1.2**	**2.4**	**0.8**	**0.6**	**0.8**	**1.3**	**30.5**	**0.4**
	Bacillus	0.2	0.6	2.2	0.5	0.1	0.3	1.3	0.0	0.2
	Staphylococcus	0.0	0.3	0.0	0.0	0.0	0.0	0.0	9.4	0.0
	Alicyclobacillus	0.0	0.0	0.0	0.0	0.0	0.0	0.0	7.3	0.0
	Streptococcus	0.0	0.0	0.1	0.0	0.0	0.0	0.0	8.9	0.0
Bacteroidia		**2.6**	**7.2**	**4.6**	**2.0**	**3.7**	**5.3**	**3.2**	**3.4**	**0.9**
	Blvii28	2.5	7.2	4.4	2.0	3.6	5.2	3.2	3.3	0.9
Cytophagia	***Adhaeribacter***	**0.0**	**0.0**	**0.0**	**0.0**	**0.0**	**0.0**	**0.0**	**1.2**	**0.0**
Clostridia		**0.9**	**0.8**	**1.0**	**0.5**	**0.4**	**0.6**	**0.0**	**5.6**	**1.0**
	Anaerococcus	0.0	0.0	0.0	0.0	0.0	0.0	0.0	5.5	0.9
Coriobacteriia	***Atopobium***	**0.0**	**0.0**	**0.0**	**0.0**	**0.0**	**0.0**	**0.0**	**2.7**	**0.0**
Deinococci		**0.0**	**0.0**	**2.0**	**0.0**	**0.0**	**0.0**	**0.0**	**0.0**	**0.0**
	Thermus	0.0	0.0	1.8	0.0	0.0	0.0	0.0	0.0	0.0
TM7	***TM7***	**0.0**	**0.0**	**0.0**	**0.0**	**0.0**	**0.0**	**8.0**	**0.0**	**0.0**
Flavobacteriia	***Cloacibacterium***	**0.3**	**0.3**	**2.1**	**0.3**	**0.4**	**1.5**	**0.0**	**0.0**	**0.5**
Acidimicrobiia	***Unclassified***	0.0	0.0	0.0	0.0	0.0	0.0	1.9	0.0	0.0
**Class <1% Abundance/ Unclassified**		**0.8**	**1.3**	**1.3**	**0.6**	**0.5**	**0.5**	**1.1**	**3.7**	**0.0**
**Total Proportion SRB**		**82.1**	**70.4**	**69.1**	**84.5**	**80.8**	**71.6**	**10.5**	**0.2**	**82.8**
**Total Proportion SOB**		**0.0**	**0.0**	**0.0**	**0.0**	**0.0**	**0.0**	**0.0**	**0.0**	**0.0**
**Total Proportion Gram Positive**		**2.1**	**2.7**	**9.2**	**2.1**	**1.9**	**1.8**	**13.5**	**86.8**	**1.4**
**Total Proportion Acidophilic**		**0.0**	**0.1**	**0.4**	**0.0**	**0.0**	**0.0**	**47.2**	**0.0**	**0.0**

Supplementary Table 5. C-Amended treatment samples as proportional abundance (%) of taxa of representative class (bold) and genera from 16S rRNA gene sequences from surfaces and planktonic sample after 3-weeks within the C-Amended reactor. Potential sulfur-oxidizing genera (SOB), sulfur-reducing genera (SRB), acidophilic genera, and gram-positive genera are highlighted.
