# Supplementary material for: Mineral Ecology: Surface Specific Colonization and Geochemical Drivers of Biofilm Accumulation, Composition, and Phylogeny
Source: Front Microbiol. 2017 Mar 28;8:491. doi: 10.3389/fmicb.2017.00491 (PMC5368280; doi:10.3389/fmicb.2017.00491)
Supplement: Supplementary file 7 [file Image1.PDF]

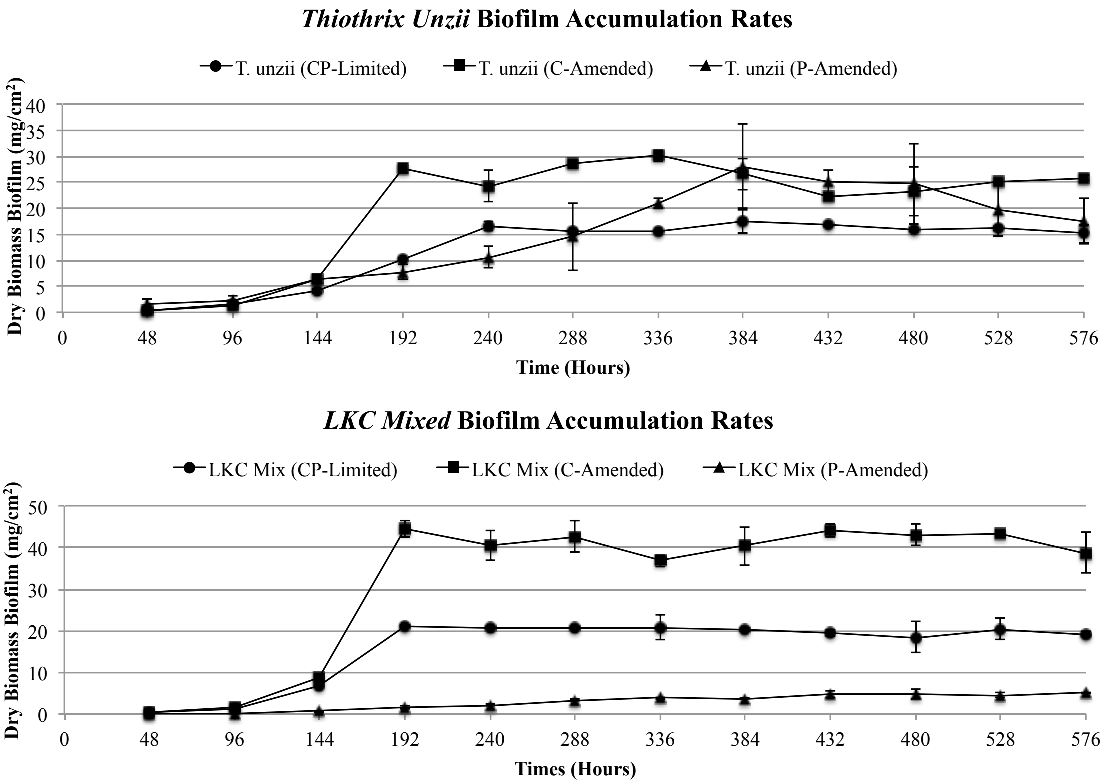

Supplementary Figure 1. Growth curves for biofilm accumulated on limestone surfaces in pure culture (*Thiothrix unzii*) and *LKC mixed culture* reactors under CP-Limited, C-Amended, and P-Amended media treatments. Error bars represent duplicate experiments.
