# Supplementary material for: Mineral Ecology: Surface Specific Colonization and Geochemical Drivers of Biofilm Accumulation, Composition, and Phylogeny
Source: Front Microbiol. 2017 Mar 28;8:491. doi: 10.3389/fmicb.2017.00491 (PMC5368280; doi:10.3389/fmicb.2017.00491)
Supplement: Supplementary file 8 [file Image2.PDF]

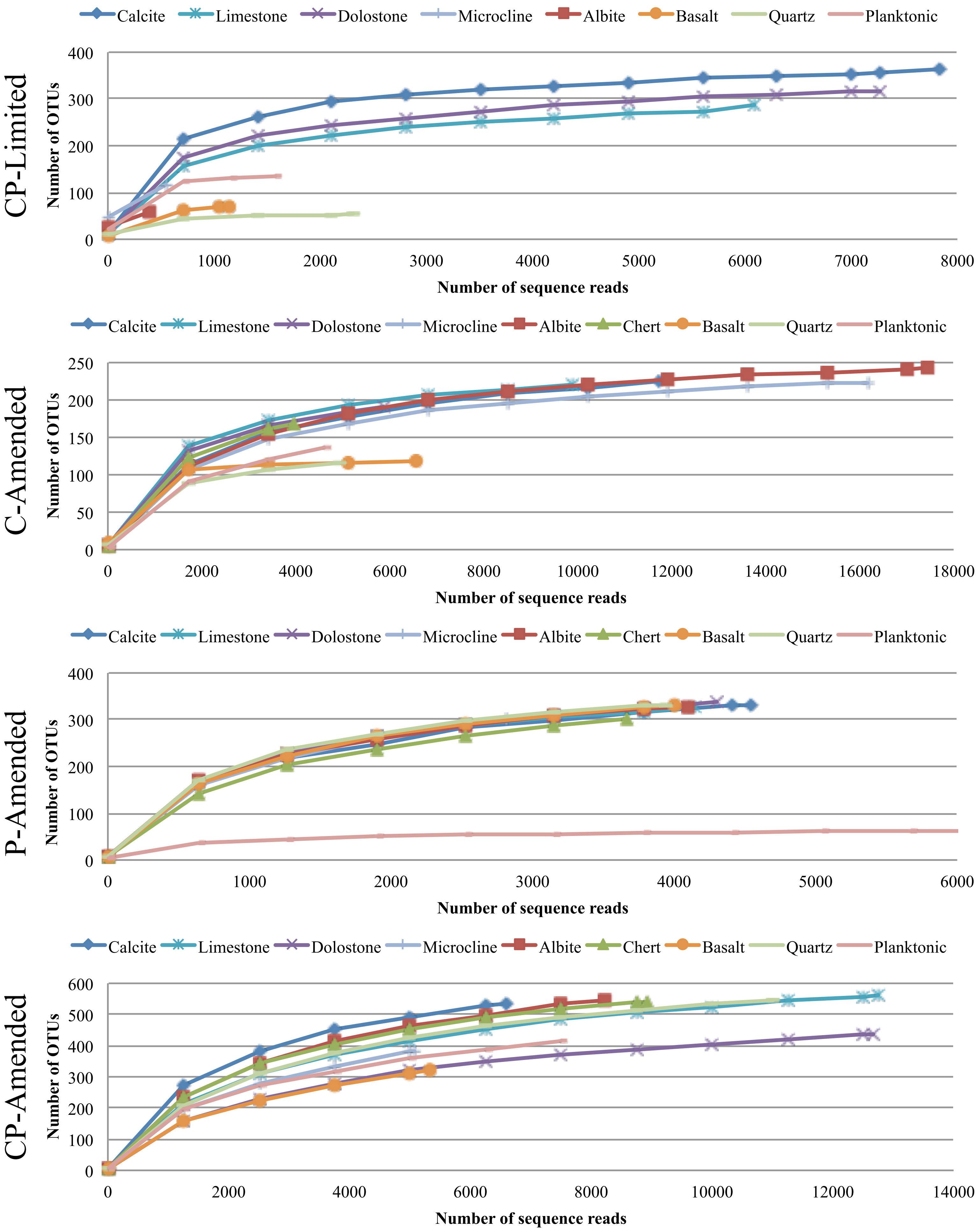

Supplementary Figure 2. Rarefaction curves of 16S rRNA sequences. OTUs were defined at 97% similarity cutoff. The figure depicts the comparison between samples from all four treatments.
